# Comparing the impact of older age on outcome in chronic kidney disease of different etiologies: a prospective cohort study

**DOI:** 10.1007/s40620-018-0529-8

**Published:** 2018-09-05

**Authors:** Maharajan Raman, Darren Green, Rachel J. Middleton, Philip A. Kalra

**Affiliations:** 1 Vascular Research Group, Department of Renal Medicine, Salford Royal NHS Foundation Trust, Stott Lane, Salford M6 8HD UK; 2 Faculty of Biology, Medicine and Health, University of Manchester, Manchester, UK

**Keywords:** CKD, Older age, Survival

## Abstract

**Background:**

In older patients with chronic kidney disease (CKD), the risk of progression to end stage renal disease and cardiovascular death both differ compared to younger patients. This likely reflects differences in case mix and co-morbid burdens. We sought to establish the extent to which age itself is an independent biomarker of adverse outcome in CKD.

**Methods:**

This was an analysis of the Salford Kidney Study, a prospective, longitudinal, observational study of 2,667 patients with eGFR < 60 ml/min/1.73 m^2^. Patients were divided into four age groups (< 55, 55–65, 65–75 and > 75 years). Within group adjusted hazard ratios for death in older compared to younger patients were calculated for different primary renal diseases. A competing risk model of death and renal replacement therapy (RRT) as outcomes was performed.

**Results:**

The median age of the cohort was 67.1 years [interquartile range (IQR): 55.6–75.3] and median eGFR 30.8 ml/min/1.73 m^2^ (IQR: 20.6–43.2). Follow up was 3.5 ± 2.9 years. Overall, the adjusted HR for death in patients aged > 75 years compared to those < 55 years was 4.4 (95% CI 3.4–5.9), p < 0.001. The HR for death differed between primary renal diseases and CKD stages. In diabetic nephropathy, the HR was 3.0 (1.8–5.3, p < 0.001), in glomerulonephritis the HR was 12.2 (5.6–25.5, p < 0.001). The cumulative incidence of RRT was < 0.1 at 10 years for patients > 75 years, compared with 0.50 in those < 55 years. Death was more likely at 20 months in those aged 75 years or older (0.17) than at 10 years in those aged < 55 years (0.10).

**Conclusion:**

This study demonstrates that the risk associated with older age shows significant variability between primary renal diseases. This is whilst acknowledging that observational studies carry the risk of hidden bias not adjusted for in the statistical model.

**Electronic supplementary material:**

The online version of this article (10.1007/s40620-018-0529-8) contains supplementary material, which is available to authorized users.

## Introduction

The general population is ageing [1], and the prevalence of stages 3 to 5 CKD amongst adults is increasing [2]. The risk of developing CKD increases with age. The NEOERICA project showed that among 65,126 subjects aged 18 to > 85 years, the proportion of subjects with CKD stages 3 to 5 was 6.9% between ages 55–64 years and 44.7% in people > 85 years [3]. CKD in older patients can be unrecognized but also may be inappropriately managed, for example with a failure to address secondary preventive measures [4]. It is well known that subjects with CKD have a high cardiovascular risk [5, 6]. Older CKD patients differ compared to younger patients in that their risk of progression to end stage renal disease (ESRD) is lower than the risk of cardiovascular death [7, 8], whereas the opposite is true in younger patients. Older patients also demonstrate slower CKD disease progression. This is likely to reflect the different case mix of primary renal diseases found across different age groups, and the greater co-morbid burden of advancing age [9–11].

We sought to establish the extent to which age itself is a biomarker of adverse outcome in CKD, independent of these factors, and to determine whether any apparent increased risk associated with older age differs between primary renal diseases, and between different CKD stages at presentation. This would provide nephrologists with more individualized risk stratification information based on age, eGFR and primary renal disease. To achieve this, the primary aim of the study was to determine the relative risk of death in older compared to young patients with CKD, specifically whether the greater risk of death associated with older age varies between primary renal diseases. The secondary aim was to explore the likelihood of older versus younger patients reaching end stage kidney disease in the context of an increasing risk of death with older age, using a competitive risk approach.

## Methods

This was a sub-study of the Salford Kidney Study, a single centre, prospectively collected, observational study of outcomes in Chronic Kidney Disease in the United Kingdom, recruiting since March 2002. Patients who are referred to the Nephrology Secondary Care outpatient clinic at Salford Royal NHS Foundation Trust, or admitted to the Nephrology inpatient ward, are approached for inclusion in the study, and are enrolled if written, informed consent is gained. The inclusion criteria are: age ≥ 18 years at the time of consent; eGFR < 60 mL/min/1.73 m^2^ (calculated using the creatinine-based 4 variable MDRD equation); and able to give written, informed consent for participation. Patients with acute kidney injury, functioning renal transplant, or already established on dialysis are enrolled into SKS but were excluded from this analysis. The study complies with the declaration of Helsinki and local ethical approval has been obtained (current REC reference 15/NW/0818).

Patients undergo an annual review including detailed clinical phenotyping and event reporting. Phenotype and outcome data are collected from patient self-reporting, Hospital Electronic Patient Records, primary care records, and mortality data from the Office for National Statistics. Patients with a previous history of myocardial infarction or coronary artery revascularization (including bypass graft), or a history of medically managed angina, were classified as having coronary artery disease (CAD). Patients with previous history of ischemic or haemorrhagic stroke, or transient ischemic attack (TIA), were classified as having cerebrovascular disease (CVD). Patients with symptoms of claudication, presence of distal ischemic ulcers, amputation due to distal ischemia, or who had undergone a peripheral arterial revascularization procedure, were classified as having peripheral vascular disease (PVD). Patients with clinical diagnosis of heart failure, with no other alternative explanation for the patient’s symptoms of breathlessness, or an echocardiogram showing evidence of either left ventricular ejection fraction (LVEF) < 50% or meeting diastolic dysfunction criteria, were classified as having congestive cardiac failure (CCF).

The study population of 2667 patients was divided in to four age groups: < 55 years, 55–65 years, 65–75 years and > 75 years. Baseline demographic data, primary renal disease, co-morbidities, medications, and laboratory data were compared between patients of different ages. The distribution of variable was assessed using the Shapiro–Wilk test. Normally distributed variables were reported as mean ± standard deviation (SD), skewed variables were reported as median with interquartile range (IQR), and categorical variables were reported as percentages. To assess the difference in baseline characteristics between different age groups, analysis of variance (ANOVA) or Kruskal–Wallis (depending on the distribution of the variable) and Chi square tests, were used for continuous and categorical variables, respectively.

We calculated hazard rations for death in older patients compared to the reference group of patients aged < 55 years in the entire study population, and in individual primary renal diseases. Analysis was performed using a multi-variate Cox proportional hazard model, with variables which were statistically significantly associated with outcome on univariate analysis included alongside age group. Analysis of renal replacement therapy and death in a competitive risk approach was performed using the “cmprsk” package within R [12, 13].

Follow up started on the date of first study visit, through to death (all cause) or 31st May 2015. In the competitive risk model, RRT was defined as the initiation of dialysis (either peritoneal or haemodialysis), or transplantation. Patients for conservative care, not RRT were censored when eGFR was first < 10 mL/min/1.73 m^2^.

Specific primary renal diseases chosen for sub-group analysis were hypertension, atherosclerotic renovascular disease (ARVD), diabetes and glomerulonephritis (GN). A pragmatic approach to categorisation was used, in that primary diagnoses were often presumed rather than biopsy proven and only one diagnosis was assigned to each patient.

## Results

2667 patients were included in the analysis. The median age of the cohort was 67.1 years [interquartile range (IQR): 55.6–75.3] and median eGFR was 30.8 ml/min/1.73 m^2^ (IQR: 20.6–43.2). 62% were men. The cohort divided into different age groups as follows: 24% (n = 641) were aged < 55 years; 19% (n = 504) were aged 55–65 years; 30% (n = 812) were aged 65–75 years, and 27% (n = 710) were aged > 75 years.

### Baseline characteristics

A detailed outline of baseline characteristics comparing different age groups is found in Table 1. Over the age of 75 years, hypertension and atherosclerotic renovascular disease (ARVD) were the commonest causes of CKD (20% and 21% respectively). In the youngest age group (< 55 years), glomerulonephritis (GN) and polycystic kidney disease (ADPKD) were the commonest causes of CKD (26% and 12% respectively). Hypertension and ARVD accounted for just 8% of CKD in this younger group. The cause of GN differed between age groups. The most common cause of GN in patients under 55 years was IgA nephropathy (45%) followed by FSGS (25%), and membranous disease (12%). The most common cause in patients aged 55–65 years was also IgA nephropathy (35%) followed by FSGS (22%) and membranous disease (18%). In patients aged 65–75 years, IgA nephropathy (41%) and membranous disease (20%) remained very common but ANCA mediated vasculitis was more frequently seen than FSGS (15% versus 13%). In patients aged > 75 years, membranous disease was more common (33%) than IgA nephropathy (31%). FSGS was seen in 15% of GN patients > 75 years, and ANCA vasculitis 8%.

**Table 1:** Baseline characteristics, overall and comparing different age groups

	Overall (n = 2667)	< 55 years (n = 641)	55–65 years (n = 504)	65–75 years (n = 812)	> 75 years (n = 710)
Age (years)*	67.1 (55.6–75.3)	45.5 (37.8–50.3)	60.7 (58.3–63.1)	70.2 (67.5–72.3)	79.5 (77.1–82.5)
Mean SBP*	138 (124–152)	130 (119–142)	138 (123–150)	140 (128–155)	142 (127–157)
Male%	62.1	56.3	64.1	64	64.1
Smoker%	66.7	54	67.3	72.3	71.4
Primary renal disease					
Hypertension%	12.8	5.1	10.1	13.9	20.3
ARVD%	12.2	3	7.3	14.8	21.1
Diabetes%	16.8	14.7	20.2	20.1	12.7
Obstruction%	1.4	0.8	1.4	1.4	2.1
GN%	15.9	25.7	20.6	12.6	7.6
Pyelonephritis%	5.8	12	4.8	4.1	3
ADPKD%	5.1	12.6	5	2.6	1.1
Other%	15.7	17.5	18.1	16.1	12
Unknown%	14.1	8.3	12.5	14.4	20.1
Comorbidities					
CAD%	28.6	7.2	23.2	36.3	43.5
PVD%	21.6	11.5	22.4	26.2	25
CVD%	14.4	4.8	11.4	19.7	20.3
CCF%	19.4	8	14.3	23.7	28.6
Diabetes%	32.4	19.2	37.2	39	33.2
Medication					
N anti-HT*	2(1–3)	2(1–3)	2 (1–3)	2 (2–3)	2 (2–3)
RAS-i%	62.1	72.5	65.3	62.2	53.7
ESA%	13.7	11.7	11.7	14.2	16.6
Statin%	60.2	44.6	62.1	70.2	61.6
Beta blocker%	38.1	31.8	36.5	42.3	40
CCB%	53	45	56.4	56.5	54.2
Laboratory					
eGFR*	30.8 (20.6–43.2)	34.3 (21.7–48.4)	33.1 (21.2–46.9)	31.7 (20.8–41.8)	27.5 (19.6–37.1)
Haemoglobin (g/l)^£^	122 ± 21	125 ± 19	124 ± 17	123 ± 16	120 ± 16
uPCR (g/mmol)*	26 (11–84)	46 (15–139)	27 (11–85)	20 (10–65)	21 (10–56)
PTH (ng/l)*	60 (33–110)	56 (31–111)	57 (34–101)	61 (37–105.7)	83 (48–132)
Albumin (g/l)*	42 (39–45)	43 (39–45)	43 (40–45)	42 (39–44)	42 (39–44)
Phosphate(mmol/l)*	1.12 (0.98–1.29)	1.15 (0.97–1.33)	1.11 (0.96–1.31)	1.11 (0.97–1.28)	1.13 (1.0–1.26)
Calcium (mmol/l)*	2.28 (2.2–2.37)	2.27 (2.18–2.36)	2.27 (2.18–2.36)	2.28 (2.2–2.37)	2.28 (2.19–2.37)
Cholesterol*	4.5 (3.7–5.3)	4.9 (4.1–5.8)	4.4 (3.7–5.4)	4.3 (3.6–5.1)	4.2 (3.5–4.9)

*SBP* systolic blood pressure, *ARVD* atherosclerotic renovascular disease, *GN* Glomerulonephritis, *ADPKD* autosomal dominant polycystic kidney disease, *CAD* coronary artery disease, *PVD* peripheral vascular disease, *CVD* cerebrovascular disease, *CCF* congestive cardiac failure, *N anti-HT* number of anti-hypertensive medications, *RAS-I* renin-angiotensin system inhibitors, *ESA* erythropoietin stimulating agent, *CCB* calcium channel blocker, *eGFR* estimated glomerular filtration rate, *uPCR* urine protein creatinine ratio, *PTH* parathyroid hormone

*Median (interquartile range)

^£^Mean ± standard deviation

With increasing age, there was significant accumulation of co-morbidities. Comparing the age groups > 75 years and < 55 years, 43.5% versus 7.2% had coronary artery disease (CAD), 25% versus 11.5% had peripheral vascular disease (PVD), 20.3% versus 4.8% had cerebrovascular disease (CVD), 28.6 versus 8% had congestive cardiac failure (CCF), and 33.3% versus 19.2% had diabetes.

Only 53.7% of patients > 75 years were prescribed renin angiotensin system inhibitors (RAS-i), compared to 72.5% of the patients aged < 55 years. Details of this and other prescribed medication are shown in Table 1.

Table 1 also details baseline laboratory parameters between age groups. The median eGFR for the > 75 years group was the lowest, 27.5 ml/min/1.73 m^2^ (IQR: 19.6–37.1). This compared with 34.3 ml/min/1.73 m^2^ (IQR: 21.7–48.4) in the < 55 years group. All of the parameters in Table 1 (including demographics, co-morbidities, primary renal disease, medication, laboratory parameters) differed between age groups at a level p < 0.01, except for calcium (p = 0.144).

The mean follow up of the cohort was 3.5 ± 2.9 years. During follow up, 34% (n = 897) of the cohort had died and 18% (n = 474) had begun RRT. Mortality per 1000 patient-years was the highest in the age group > 75 years (175.1) and lowest in the age group < 55 years (29.9). Coupled with this, the number of patients commencing RRT per 1000 patient-years was lowest in the age group > 75 years (22.1) and highest in the age group < 55 years (86.8). Full details of event rates per 1000 patient years for death and ESRD for the whole population and each primary disease diagnosis are found in Table 2.

**Table 2:** Events rates per 1000 patient years for all-cause mortality and commencing renal replacement therapy (RRT), divided according to age

Age group (years)	Total number	Mortality (n = 897)	RRT (n = 474)
< 55	641	29.9	86.8
55–65	504	53.2	50.8
65–75	812	120.6	40.8
> 75	710	175.1	22.1

### Survival analysis

Within the whole study population, the factors which were significantly associated with mortality on univariate Cox proportional hazards regression analysis were male gender, SBP, smoking, baseline eGFR, CAD, CVD, PVD, CCF and diabetes. These parameters were entered into a multivariate model along with age group, with age < 55 years as the reference group. The adjusted hazard ratio (HR) for death in the age group 55–65 years compared to age < 55 years was 1.41 (95% CI 1.03–1.92), p = 0.030, in the age group 65–75 years was 2.83 (2.15–3.72), p < 0.001 and in the age group > 75 years was 4.46 (3.39–5.87), p < 0.001.

In a multivariate Cox model for all-cause mortality in specific primary renal diseases (Table 3), the HR for death increased with age in all diseases. However, the HR for death in patients aged > 75 years (relative to < 55 years) varied markedly between diseases. For patients with glomerulonephritis, the HR for death in those aged > 75 years was 12.2 (95% CI 5.6–26.5), p < 0.001, compared to the reference group. For hypertension the HR was 10.1 (95% CI 2.9–34.1), p = 0.001. In ARVD and diabetes, the HR were much lower [ARVD HR 3.4 (95% CI 1.2–9.5) p = 0.02, diabetes HR 3.0 (95% CI 1.8–5.3) p < 0.001].

**Table 3:** A comparison of adjusted hazard ratios for death in older compared to younger patients across different primary diseases

	Hypertension				ARVD				Diabetes				Glomerulonephritis			
Age	N	HR	95% CI	P	N	HR	95% CI	P	N	HR	95% CI	P	N	HR	95% CI	P
Death																
< 55*	31	1.00			19	1.00			92	1.00			161	1.00		
55–65	51	0.8	0.1–3.9	0.751	35	2.2	0.7–6.8	0.161	99	1.1	0.6–1.9	0.738	100	1.5	0.6–3.4	0.335
65–75	109	5.7	1.7–19.8	0.006	116	3.3	1.2–9.1	0.024	160	1.7	1.0–2.8	0.047	100	4.9	2.4–9.9	< 0.001
> 75	140	10.1	2.9–34.1	< 0.001	140	3.4	1.2–9.5	0.020	83	3.0	1.8–5.3	< 0.001	51	12.2	5.6–26.5	< 0.001

*RRT* renal replacement therapy, *ARVD* atherosclerotic renovascular disease, *N* number, *HR* hazard ratio, *CI* confidence interval

### Competitive risk analysis of death and renal replacement therapy

There was a striking difference in incidence of RRT between younger and older patients. For those aged < 55 years, the cumulative incidence at 5 years was 0.36, and 0.50 at ten years. By contrast, RRT was uncommon in older patients (0.08 at 5 years, and 0.09 at 10 years for age 75 years or more).

Death was uncommon in the youngest age group. The cumulative incidence was 0.05 at 5 years and 0.10 at 10 years. In the age group 55–64 years, RRT remained more frequent as an outcome that death (0.20 versus 0.15 at 5 years, 0.33 versus 0.24 at 10 years). In the two older age groups, however, death was more likely than RRT.

The cumulative incidence for death at 5 years follow up in the age groups 65–74 and 75 or more years was 0.34 and 0.51 respectively. At 10 years the cumulative incidences were 0.57 and 0.83 respectively for these groups. Indeed, death was more common by 20 months in those aged 75 years or older (0.17) than at 10 years in those aged < 55 years (0.10).

In analysis of primary disease specific patterns of outcome, the likelihood of RRT was much higher for younger than older patients in diabetic nephropathy, hypertension, and ARVD, following similar patterns to that seen in the whole population analysis. The glomerulonephritis group followed a different pattern with the cumulative incidence of RRT being broadly similar across all age groups. For those under 55 years with glomerulonephritis, the cumulative incidence of RRT at 5 years was 0.28, for age 55–64 years was 0.22, for age 65–74 years was 0.17, and for patients aged 75 years or older was 0.20. Table 4 displays the likelihood of death and RRT at 5 years follow up in a competing risk model, divided by primary renal disease and age on presentation.

**Table 4:** Cumulative incidence of death and renal replacement therapy (RRT) at 5 years follow up for patients with chronic kidney disease in a competing risk model

Age (years)	All	GN	DM	HT	ARVD
Death					
Under 55	0.05	0.05	0.07	0.00	0.06
55 to 64	0.15	0.08	0.21	0.08	0.32
65 to 74	0.34	0.32	0.38	0.28	0.51
75 plus	0.51	0.43	0.70	0.50	0.52
RRT					
Under 55	0.36	0.28	0.50	0.37	0.52
55 to 64	0.20	0.22	0.27	0.20	0.08
65 to 74	0.16	0.17	0.22	0.11	0.07
75 plus	0.08	0.20	0.06	0.06	0.06

Results are divided by primary renal disease and age on presentation

*All* all diagnoses, *GN* glomerulonephritis, *DM* diabetic nephropathy, *HT* hypertension, *ARVD* atherosclerotic renovascular disease

**Fig. 1:**
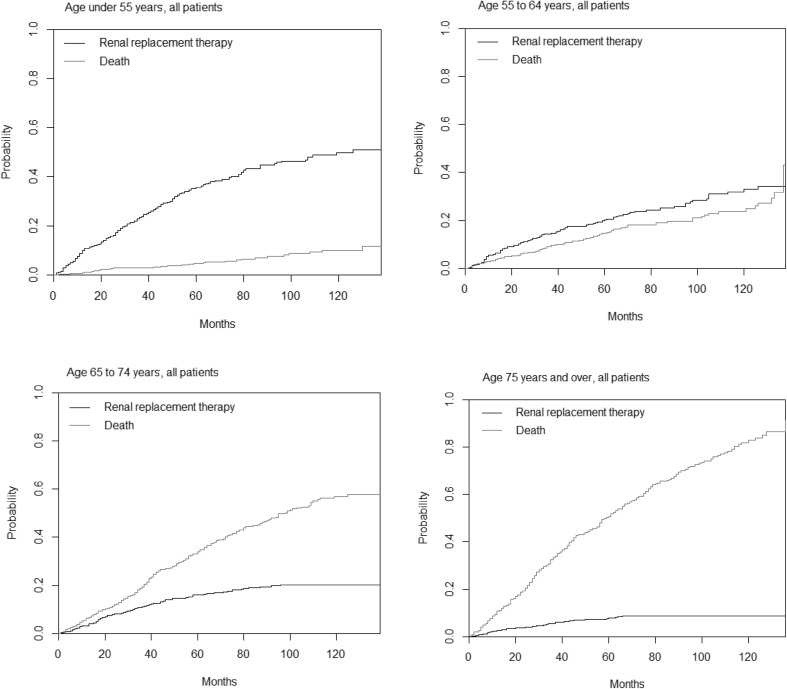
Cumulative incidence of death and renal replacement therapy in a competitive risk model, in patients with chronic kidney disease divided into age groups

The likelihood of death was low in all diagnoses for the youngest patient group, the cumulative incidence at 5 years being no higher than 0.07 for any given diagnosis. Diabetic nephropathy was associated with the highest likelihood of death in all age groups above 55 years at 5 years follow up (cumulative incidence 0.21 for age 55–64 years, 0.38 for age 65–74 years, 0.70 for patients aged 75 years or older). Cumulative results for death and RRT as competitive risks for patients of different age groups are displayed in Fig. 1. Full details of risk for both end points at all time points up to 10 years for all diagnoses can be found in online table 1.

**Fig. 2:**
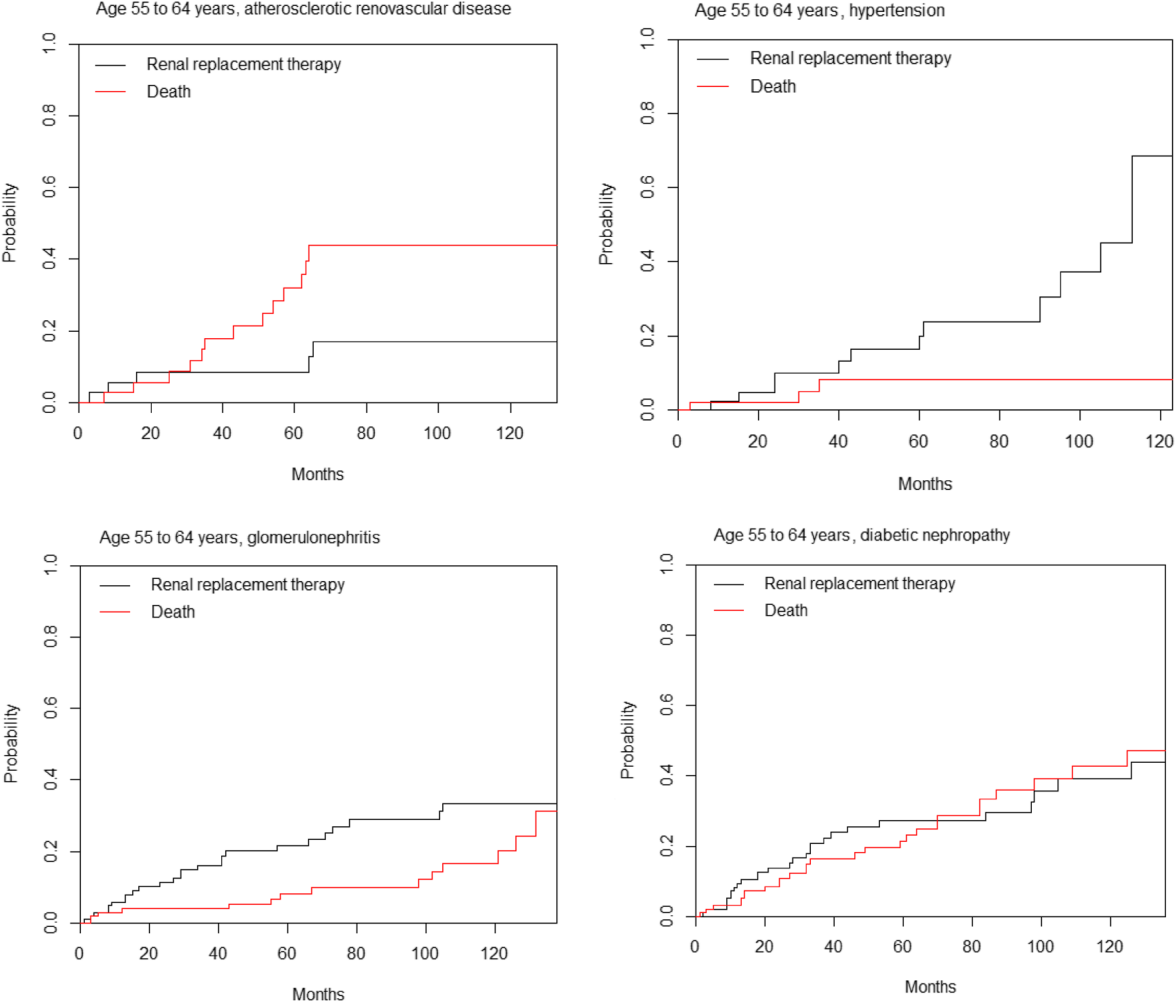
A comparison of the cumulative likelihood of death and renal replacement therapy in different primary renal diseases, for patients aged 55–64 years

The age group in which death became a cumulatively more likely outcome that RRT differed between primary diseases (Fig. 2). In the whole population, this occurred in the age group 65–74 years. For ARVD, death was more likely than RRT during follow up for all age groups above age 55 years. For glomerulonephritis patients, death was more likely as an end point in patients aged 65 years or older. For those aged 55–64 years, RRT was more likely than death at 5 years, but not at 10 years. In hypertension, death became more likely than RRT in the age 65 years or older group. For diabetes, death and RRT were equally likely at all points during follow up for those aged 55–64 years. Again, death became more likely than RRT at all stages of follow up in those aged 65 years or more on presentation.

## Discussion

A key finding of his study is that the relative risk of death in older compared to younger CKD patients differs markedly between primary renal diseases. Older patients with glomerulonephritis have the highest adjusted risk for death compared to younger patients with the same diagnosis, and ARVD the lowest. Interestingly, the absolute mortality rate (events per 1000 patient years) is highest in the ARVD group. This likely reflects the significant impact of cardiovascular co-morbidities on outcomes. The marked variation in mortality between age groups in patients with GN very likely reflects that this is not an homogenous diagnostic population. The cause of GN was most likely to be membranous nephropathy in the oldest patients, compared with IgA nephropathy in all other age groups. Membranous nephropathy can carry a worse prognosis, particularly when associated with other diseases such as malignancy.

Alongside this finding, and as expected, older patients have a very low likelihood of reaching end stage renal disease compared to younger patients. The progression to renal failure due to diabetes in older people has been described before [14]. Hemmelgran et al. in a study of 10,184 community dwelling older people (> 66 years) showed an overall slow rate of decline in eGFR in this age group but also highlighted that subjects with diabetes had the fastest decline in the 2 year follow up period. The age adjusted decline in eGFR per year (95% CI) for female and male diabetics was 2.1 (1.8–2.5) and 2.7 (2.3–3.1) ml/min/1.73 m^2^/year respectively, compared to women and men without diabetes, which were 0.8 (0.6-1.0) and 1.4 (1.2–1.6) ml/min/1.73 m^2^/year respectively [15]. This is low compared to the average decline of 7.5 ml/min/year in subjects with CKD described by the United States Renal Data System (USRDS) [16]. Although in our study we showed that diabetes had a low risk of progression to RRT we did not evaluate the rate of decline in eGFR which influences the progression to ESKD [17].

Several studies have shown the benefits of RAS inhibition in old people [18–20]. In our study, although the age group > 65 years had the highest accumulation of cardiovascular co-morbidities, only half of them were prescribed RAS inhibitor. This is perhaps due to the adverse events associated with these medications and the suboptimal use of secondary preventive measures in this age group described in literature [21–24].

In this prospective study of patients with CKD we have also demonstrated that, as age increases, the risk of ESKD is low compared to the risk of death. This inverse relationship between ESKD and death is consistent with previous studies [8, 25–28]. For example, O’Hare et al. in a study of 209,622 patients aged 18–100 years with CKD stages 3 to 5, showed this inverse relationship between death and ESKD [24]. In this study the adjusted HR (95% CI) for death in the age group 75–84 years, for CKD stages 3b, 4 and 5 was 2.6 (1.8–3.7), 3.1 (1.9–5.1) and 4.4 (1.6–22.7) respectively, compared to the reference group, of patients aged 18 to 44 years. The HR (95% CI) for ESKD for the same age groups, and also for CKD stages 3b to 5, were 0.1 (0.08–0.1), 0.3 (0.2–0.3) and 0.5 (0.4–0.6) respectively. In that study, only 3% of the study population was female and 86% were Caucasian. Our study has a greater proportion of female patients (38%) but is also predominantly Caucasian in origin.

Manjunath et al. in their community based study of people aged > 65 years, showed that all cause mortality increases with declining eGFR. The HR (95% CI) for all-cause mortality in patients with eGFR between 15 and 59 ml/min/1.73 m^2^, compared to an eGFR of 60 to 89 ml/min/1.73 m^2^, were 1.5 (1.1–2.1) and 1.1 (0.8–1.4) respectively [27]. De Nicola et al. showed that the risk of ESKD prevailed over death when eGFR was between 25 and 35 ml/min/1.73 m^2^ for the age group between 65 and 75 years and when the eGFR was less than 15 ml/min/1.73 m^2^ for the age group > 85 years [29]. This study also highlighted that proteinuria and increasing age are important risk factors for progressing to ESKD. In our study we did not evaluate the interaction of proteinuria and increasing age.

To conclude, the mix of comorbidities and prescribed medication, as well as the relative risk of death versus dialysis, is very different in older compared to younger CKD patients. Uniquely, this study demonstrates that the risk associated with older age shows significant variability between primary renal diseases. Glomerulonephritis demonstrates a worse outcome for older versus younger patients when compared to other diseases. Of note, eGFR was not independently associated with mortality in patients aged < 55 years.

It is interesting to note that although the elderly (> 65 years) have the highest cardiovascular risk, only 50–60% were prescribed renin angiotensin system inhibitors (RAS-i), compared to their younger counterparts. In this study we have also confirmed the inverse relationship between death and RRT with increasing age. Age specific risk profiling and management strategies may therefore be required given our ageing population.

This study is not without limitations. The risk of progression to RRT was based on baseline eGFR at study inception and did not evaluate the rate of change in eGFR with time, which is known to influence the progression to ESKD. The risk of death and CKD progression may also be influenced by proteinuria, but proteinuria was not assessed in this study. In our analysis we have used the 4 variable MDRD equation in the calculation of eGFR as was standard in clinical practice during the study follow up period. However, this method of GFR estimation is not well validated in older people.

## Electronic supplementary material

Below is the link to the electronic supplementary material.

Supplementary material 1 (DOCX 22 KB)
