# Supplementary material for: Comparing the impact of older age on outcome in chronic kidney disease of different etiologies: a prospective cohort study
Source: J Nephrol. 2018 Sep 5;31(6):931–9. doi: 10.1007/s40620-018-0529-8 (PMC6244557; doi:10.1007/s40620-018-0529-8)
Supplement: Supplementary file 1 — Supplementary material 1 (DOCX 22 KB) [file 40620_2018_529_MOESM1_ESM.docx]

Online table 1. Cumulative likelihood of death and renal replacement therapy (RRT) for patients with chronic kidney disease in a competing risk model. Results are divided by primary renal disease and age on presentation.

| **Age (years)** | **Outcome** | **Follow up time (months)** | | | | | |
| --- | --- | --- | --- | --- | --- | --- | --- |
|  |  | **20** | **40** | **60** | **80** | **100** | **120** |
| All patients | | | | | | | |
| under 55 | RRT | 0.14 | 0.26 | 0.36 | 0.43 | 0.46 | 0.50 |
| 55 to 64 |  | 0.09 | 0.15 | 0.20 | 0.24 | 0.28 | 0.33 |
| 65 to 74 |  | 0.07 | 0.12 | 0.16 | 0.19 | 0.20 | 0.20 |
| 75 plus |  | 0.04 | 0.06 | 0.08 | 0.09 | 0.09 | 0.09 |
| under 55 | Death | 0.02 | 0.03 | 0.05 | 0.06 | 0.09 | 0.10 |
| 55 to 64 |  | 0.05 | 0.10 | 0.15 | 0.18 | 0.21 | 0.24 |
| 65 to 74 |  | 0.10 | 0.23 | 0.34 | 0.44 | 0.51 | 0.57 |
| 75 plus |  | 0.17 | 0.37 | 0.51 | 0.65 | 0.74 | 0.83 |
| Glomerulonephritis | | | | | | | |
| under 55 | RRT | 0.11 | 0.21 | 0.28 | 0.39 | 0.41 | 0.46 |
| 55 to 64 |  | 0.10 | 0.16 | 0.22 | 0.29 | 0.29 | 0.33 |
| 65 to 74 |  | 0.07 | 0.12 | 0.17 | 0.22 | 0.25 | 0.25 |
| 75 plus |  | 0.06 | 0.20 | 0.20 | 0.20 | 0.20 | - |
| under 55 | Death | 0.02 | 0.02 | 0.05 | 0.07 | 0.07 | 0.07 |
| 55 to 64 |  | 0.04 | 0.04 | 0.08 | 0.10 | 0.12 | 0.17 |
| 65 to 74 |  | 0.06 | 0.21 | 0.32 | 0.36 | 0.45 | 0.53 |
| 75 plus |  | 0.16 | 0.43 | 0.43 | 0.66 | 0.70 | - |
| Diabetic nephropathy | | | | | | | |
| under 55 | RRT | 0.31 | 0.41 | 0.50 | 0.54 | 0.57 | 0.57 |
| 55 to 64 |  | 0.13 | 0.24 | 0.27 | 0.27 | 0.36 | 0.39 |
| 65 to 74 |  | 0.10 | 0.16 | 0.22 | 0.27 | 0.27 | 0.27 |
| 75 plus |  | 0.04 | 0.04 | 0.06 | 0.06 | 0.06 | - |
| under 55 | Death | 0.04 | 0.07 | 0.07 | 0.09 | 0.15 | 0.23 |
| 55 to 64 |  | 0.08 | 0.16 | 0.21 | 0.29 | 0.39 | 0.43 |
| 65 to 74 |  | 0.11 | 0.29 | 0.38 | 0.49 | 0.56 | 0.59 |
| 75 plus |  | 0.24 | 0.53 | 0.70 | 0.72 | 0.89 | - |
| Hypertension | | | | | | | |
| under 55 | RRT | 0.13 | 0.32 | 0.37 | 0.43 | 0.43 | 0.43 |
| 55 to 64 |  | 0.05 | 0.13 | 0.20 | 0.24 | 0.37 | 0.69 |
| 65 to 74 |  | 0.06 | 0.08 | 0.11 | 0.11 | 0.11 | 0.11 |
| 75 plus |  | 0.02 | 0.05 | 0.06 | 0.08 | 0.08 | - |
| under 55 | Death | 0.00 | 0.00 | 0.00 | 0.00 | 0.11 | 0.11 |
| 55 to 64 |  | 0.02 | 0.08 | 0.08 | 0.08 | 0.08 | 0.08 |
| 65 to 74 |  | 0.09 | 0.17 | 0.28 | 0.40 | 0.59 | 0.66 |
| 75 plus |  | 0.15 | 0.34 | 0.50 | 0.64 | 0.75 | - |
| Atherosclerotic renovascular disease | | | | | | | |
| under 55 | RRT | 0.16 | 0.34 | 0.52 | 0.52 | 0.52 | 0.52 |
| 55 to 64 |  | 0.08 | 0.08 | 0.08 | 0.17 | 0.17 | 0.17 |
| 65 to 74 |  | 0.03 | 0.06 | 0.07 | 0.09 | 0.09 | 0.09 |
| 75 plus |  | 0.05 | 0.05 | 0.06 | 0.06 | 0.06 | 0.06 |
| under 55 | Death | 0.06 | 0.06 | 0.06 | 0.06 | 0.06 | 0.06 |
| 55 to 64 |  | 0.06 | 0.18 | 0.32 | 0.44 | 0.44 | 0.44 |
| 65 to 74 |  | 0.15 | 0.30 | 0.51 | 0.58 | 0.67 | 0.76 |
| 75 plus |  | 0.16 | 0.36 | 0.52 | 0.67 | 0.73 | 0.82 |
